# A Bibliometric and Visualization Analysis of Metabolic Reprogramming in Cardiovascular Diseases: Trends, Key Contributors, and Future Directions from 2000 to 2024

**DOI:** 10.2174/011573403X371021250109064231

**Published:** 2025-02-07

**Authors:** Xing Chen, Liu Lin Yang, Li Xiang Li, Yan Deng

**Affiliations:** 1 Department of Ultrasound, The First Affiliated Hospital of Guangxi Medical University, Guangxi Medical University, Nanning, China;; 2 Department of Hepatological Surgery, The First Affiliated Hospital of Guangxi Medical University, Guangxi Medical University, Nanning, China

**Keywords:** Cardiovascular disease, metabolic reprogramming, bibliometrics, immune cell, tumor cells, hotspots

## Abstract

**Background:**

Metabolic reprogramming is critical in cardiovascular disease (CVD) research, affecting a variety of diseases such as myocardial damage, coronary heart disease, and atherosclerosis, and has also emerged as a therapeutic target. This study conducts a bibliometric analysis of the past 24 years to identify trends and hotspots in CVD metabolism, aiming to guide future research and inform policy.

**Methods:**

This study analyzes publications from January 1, 2000, to October 10, 2024, using the Web of Science Core Collection database. Tools like CiteSpace, VOSviewer, and SCImago Graphica were used for co-authorship, keyword, citation, and journal visualizations. Dual-map overlays and annual publication trends were examined to uncover hotspots, trends, and the progression of metabolic reprogramming in CVD.

**Results:**

This study analyzed 765 articles and reviews from 66 countries. The USA had the most publications, with the University of Milan being the most productive institution. Després, JP's team in Italy, published the most papers. The *International Journal of Molecular Sciences* had the highest publication count, while *Cardiovascular Diabetology* had the greatest citation impact. Recent research has mainly focused on the role of immune cell substrate metabolism in CVD.

**Conclusion:**

This study reveals the development trend and research characteristics of CVD metabolic reprogramming over the past 24 years, from the early focus on disease risk factors to the recent exploration of the transformation of immune cell metabolism. In the future, targeting immune cell metabolism will drive CVD therapy forward.

## 1 INTRODUCTION

Cardiovascular disease (CVD), encompassing conditions like coronary artery disease, hypertension, and heart failure, among others, arises from the complex interplay of atherosclerosis, genetic predispositions, and environmental influences. These factors contribute to elevated mortality and disability rates, significantly impacting patients' quality of life and presenting a substantial public health challenge on a global scale [1-3]. Despite some progress in understanding the underlying mechanisms, the complex pathophysiology of CVD remains incompletely elucidated. Recently, increasing attention has been directed towards the pivotal involvement of metabolic reprogramming in CVD. The heart, being a metabolically demanding organ, predominantly utilizes fatty acid oxidation for energy production. However, in CVD, there is a shift in cardiomyocyte metabolism from fatty acid oxidation to glycolysis, mirroring the “Warburg effect” observed in tumor cells [4, 5]. While this metabolic shift initially sustains energy production, it ultimately results in decreased energy efficiency, heightened oxidative stress, as well as compromised mitochondrial function, culminating in the deterioration of cardiomyocyte functionality and advancing the progression of heart failure. Oxidative stress and mitochondrial dysfunction hold pivotal states within cardiomyocyte injury and inflammatory reactions, fostering atherosclerosis and cardiac remodeling, particularly in the context of heart failure [6, 7]. Moreover, metabolic reprogramming influences immune cell function in CVD, particularly by altering macrophage metabolism. This metabolic alteration drives macrophages towards a pro-inflammatory phenotype from an initially anti-inflammatory state, intensifying local inflammation and leading to additional cardiac tissue injury. However, upon the restoration of metabolic equilibrium, macrophages promote anti-inflammatory responses and tissue repair, demonstrating a dual role in CVD progression [2, 8]. Endothelial cell metabolism also transitions from oxidative phosphorylation to glycolysis, enhancing their proliferation and resistance to apoptosis. This metabolic change contributes to vascular thickening, remodeling, and exacerbating cardiovascular pathology, further increasing the cardiac workload and worsening myocardial function [9-11]. Additionally, metabolic reprogramming affects branched-chain amino acids and ketone body metabolism, where sustained dependence on these alternative pathways may result in metabolic disturbances and exacerbate ischemia/reperfusion injury in the long term [12]. In summary, metabolic reprogramming offers potential therapeutic targets for CVD. Interventions aimed at modulating glycolysis, restoring fatty acid oxidation, or improving mitochondrial function may slow CVD progression and improve patient outcomes. Thus, investigations into metabolic reprogramming not only uncover the deeper mechanisms of CVD but also provide novel insights for personalized treatments and precision medicine.

Bibliometric analysis is an effective tool for systematically analyzing literature through statistical and quantitative methods [13, 14]. It helps reveal research trends, hotspots, and gaps in knowledge related to metabolic reprogramming in CVD while also identifying key researchers, institutions, and influential publications. As of now, there has been a lack of bibliometric analyses examining the current research landscape concerning metabolic reprogramming and CVD. Therefore, using tools such as VOSviewer, CiteSpace, and SCImago Graphica, we visualize the literature data, constructing co-occurrence networks, research hotspot maps, and collaboration networks. This strategy offers extensive insight into the research landscape of metabolic reprogramming in CVD, offering valuable theoretical support and a scientific foundation for future studies.

## 2 MATERIALS AND METHODS

### 2.1 Data Collection and Screening

Data were sourced from the Web of Science Core Collection (WOSCC), a widely used database offering key bibliometric indicators such as titles, keywords, countries, institutions, and categories [15]. The final search query was: TS=(“Metabolic reprogramming” OR “Metabolic remodeling” OR “Metabolic rewiring” OR “Reprogramming of metabolism” OR “Metabolic alterations”) and TS=(“coronary” OR “heart disease” OR “heart attack” OR “coronary disease” OR “coronary event” OR “myocardia” OR “congenital heart disease” OR “congenital heart defect” OR “heart valvular disease” OR “aortic valve” OR “mitral valve” OR “tricuspid valve” OR “heart abnormal” OR “heart malform” OR “Cardiovascular Diseases” OR “Cardiovascular Disease” OR “Disease, Cardiovascular” OR “Diseases, Cardiovascular”). We collected all publications from the WOSCC database dated from January 1, 2000 to October 10, 2024. The retrieved documents comprised articles and review articles exclusively in English. Out of the initial 801 publications, 18 non-article and non-review documents, as well as 18 non-English documents, were excluded. Ultimately, 765 records were exported, including complete information on the final selected publications and their cited references. Written informed consent was obtained from all individual participants included in this study for the publication of their personal data, images, and any accompanying materials. The consent process was conducted in compliance with ethical guidelines, and the original consent forms were retained by the corresponding author. The publication of these materials has been approved with the understanding that the data will be used exclusively for academic and research purposes.

### 2.2 Data Collection and Filtering

**Fig. (1):**
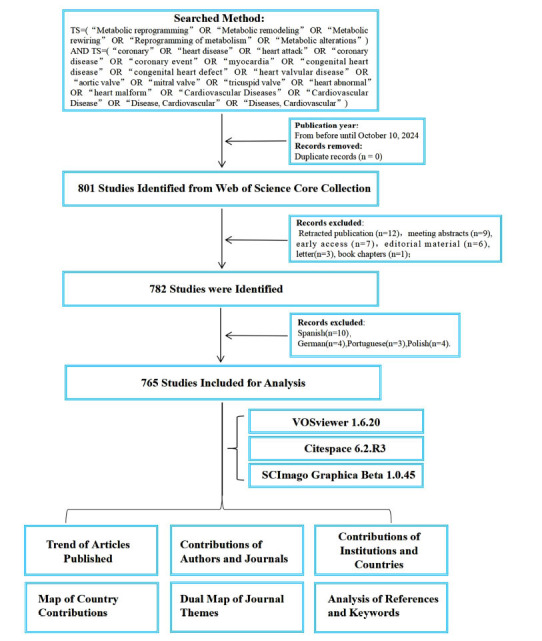
The overall workflow design for literature screening and data analysis.

In this study, the data were visualized through CiteSpace (version 6.2.R3) and VOSviewer (version 1.6.20). VOSviewer enables the construction of network maps, facilitating the examination of institutional and author co-authorship, co-citation of references and journals, as well as author co-citation patterns [16]. CiteSpace was employed to create a dual-map overlay of journals. Furthermore, the identification and discussion of sudden research hotspots emerging in specific years within the field were conducted. The yearly and total publication numbers were visualized through GraphPad Prism (version 10.2.1). The overall workflow design for literature screening and data analysis is presented below (Fig. **1**).

## 3 RESULTS

### 3.1 The Annual Publication Trend

**Fig. (2):**
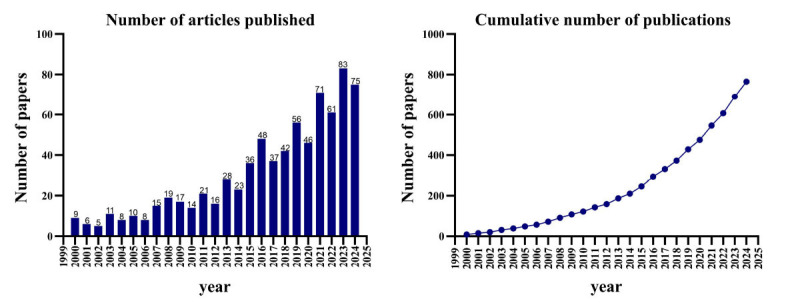
Trends in the number of published articles on metabolic reprogramming and CVD.

In this study, 765 research articles meeting the screening criteria were analyzed, authored by 5,224 researchers from 1,415 institutions and published across 409 journals in 66 countries. These studies collectively cited 53,573 references, contributed by 38,703 authors and published in 5,185 journals. From 2000 to 2006, an average of 8 papers per year focused on metabolic reprogramming and CVD, indicating a steady trend. Subsequently, the publication output gradually rose from 2007 to 2020, with a notable surge observed between 2021 and 2024, reaching a peak of 83 publications in 2023 (Fig. **2**). This increasing trend indicates the ongoing expansion of research endeavors in the future.

### 3.2 Analysis of the Most Prolific and Highly Co-cited Authors

**Fig. (3):**
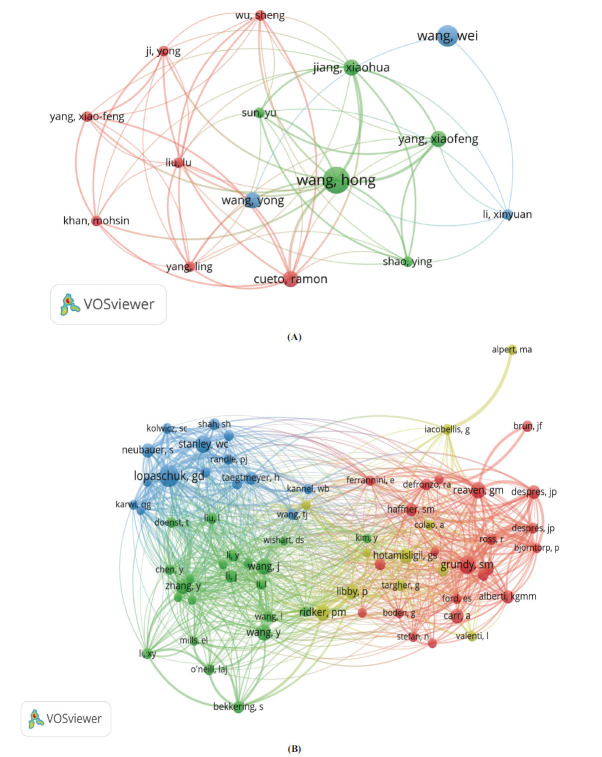
Visual maps of authors generated by VOSviewer. (**A**) Authors publish more than 2 made network analyses; (**B**) Network analysis made by more than 70 co-cited authors.

VOSviewer analysis revealed that 5,224 authors have contributed to research on metabolic reprogramming and CVD over the past 25 years (Fig. **3A**, Table **1**). Among them, 14 authors have published at least four papers. The most prolific authors are Després, JP from Canada (5 papers, only highlights the key nodes) (Fig. **3A**), Silvia Fargion from Italy, and Wang Hong from China. Després, JP also has the highest number of citations (550), followed by Bergeron, J (542 citations). Notably, Bergeron, J boasts the highest average citation rate (135.5 per paper), with Després, JP closely trailing with 110 citations per paper. Additionally, among 38,703 co-cited authors, 62 authors have a co-citation threshold exceeding 20. The co-citation network of the journal highlights Lopaschuk, GD as the most cited author (79 citations). Furthermore, Grundy, SM ranks second with 60 citations, and Stanley, WC ranks third with 295 citations (Fig. **3B**).

### 3.3 Analysis of the Countries and Institutions with the Highest Number of Publications

**Fig. (4):**
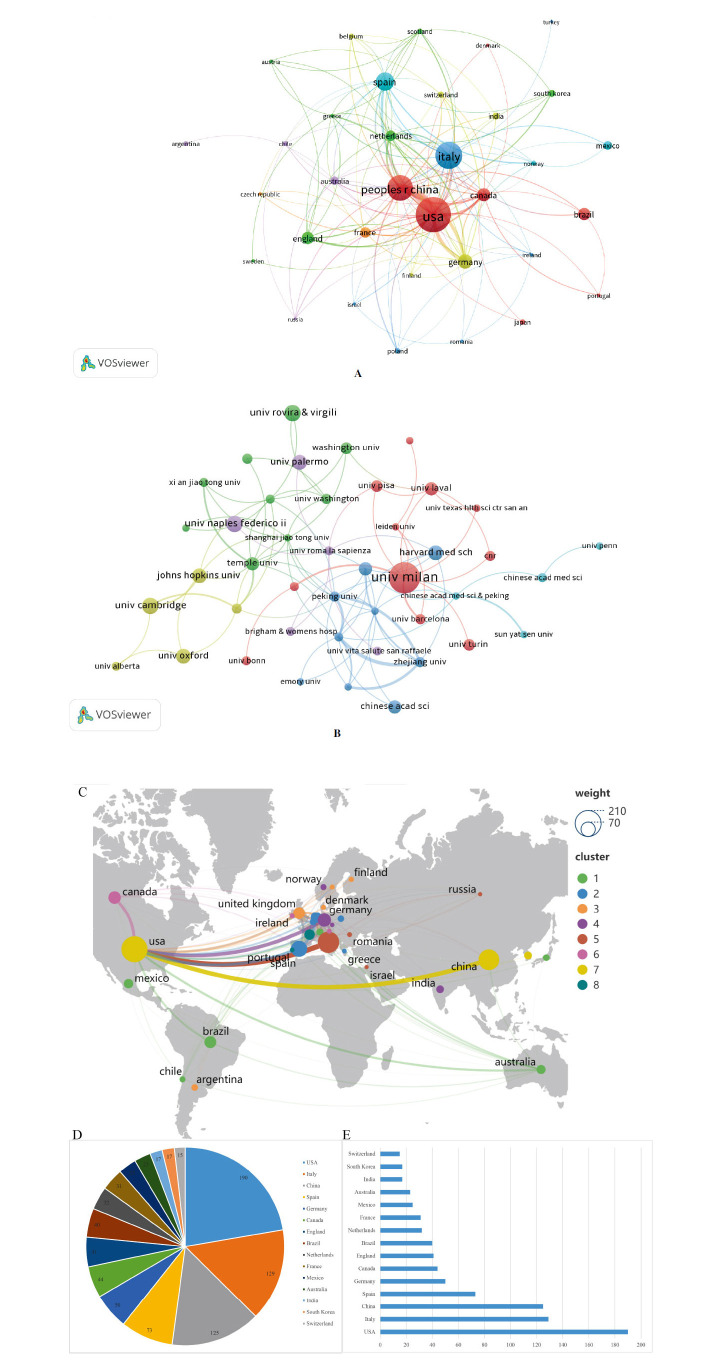
Visual representation depicting the number of papers published by countries and co-cited institutions. (**A**) Network map of publication volume by country, including country with a minimum of five published articles; (**B**) Co-cited institutions network map generated by VOSviewer, featuring institutions with at least five publications; (**C**) Geographic distribution map of the country generated by Scimago Graphica; (**D**) Pie chart of the number of publications by country; (**E**) Bar chart of the number of publications by country.

The exploration of metabolic reprogramming and CVD spans 66 countries, with a network map of 35 countries that published five or more papers (Fig. **4A** and Table **2**). The U.S. emerges as the largest node with the highest publication count (190 papers), indicating its leading role, followed by Italy (129 papers) and China (125 papers), underscoring their significant positions. In total citation count, the United States leads (11,183 citations), trailed by Italy (5,875) and Canada (3,920). Canada boasts the highest average citation rate (89.09), with Germany (64.82) and the United Kingdom (62.93) in close pursuit. Moreover, an institutional collaboration network was generated through VOSviewer, focusing on institutions that have published five or more papers between 2000 and 2024, resulting in the identification of 57 institutions (Fig. **4B** and Table **S1**). Leading roles are assumed by the University of Milan, Instituto de Salud Carlos III (ISCIII), University of Cambridge, University of Naples Federico II, and Rovira i Virgili University within the network. The University of Milan secures the top spot in publication count (21 papers), closely followed by ISCIII (12 papers), while the University of Cambridge, University of Naples Federico II, and Rovira i Virgili University each contribute 11 papers. Laval University has the highest total citations (2,235), followed by the University of Milan and the University of Cambridge, and also achieves the highest average citation rate (248.33).

Global scientific collaborations are predominantly concentrated in North America and Europe, with the United States and China emerging as the most prominent nodes. The distribution indicates a higher volume of publications originating from these regions, while Asia contributes relatively fewer studies (Fig. **4C**). The distribution of publications across countries is visually represented through pie and bar charts, showcasing the U.S. as the leading contributor with 190 papers, followed by Italy with 129 and China with 125. Spain (73 papers), Germany (50), and Canada (44) are also notable contributors. Other prominent contributors include the U.K. (41 papers), Brazil (40), and the Netherlands (32), while France (31) and Mexico (25) have moderate outputs. Australia, India, South Korea, and Switzerland contribute 23, 17, 17, and 15 papers, respectively (Figs. **4D**, **E**). These data underscore the central role of developed countries, particularly the U.S. and Italy, in advancing global research in this field, with developing countries like China, Brazil, and Mexico also playing essential roles.

### 3.4 Journals with the Highest Number of Publications and the Most Cited Journals, as well as Analysis of Citing and Cited Journals

**Fig. (5):**
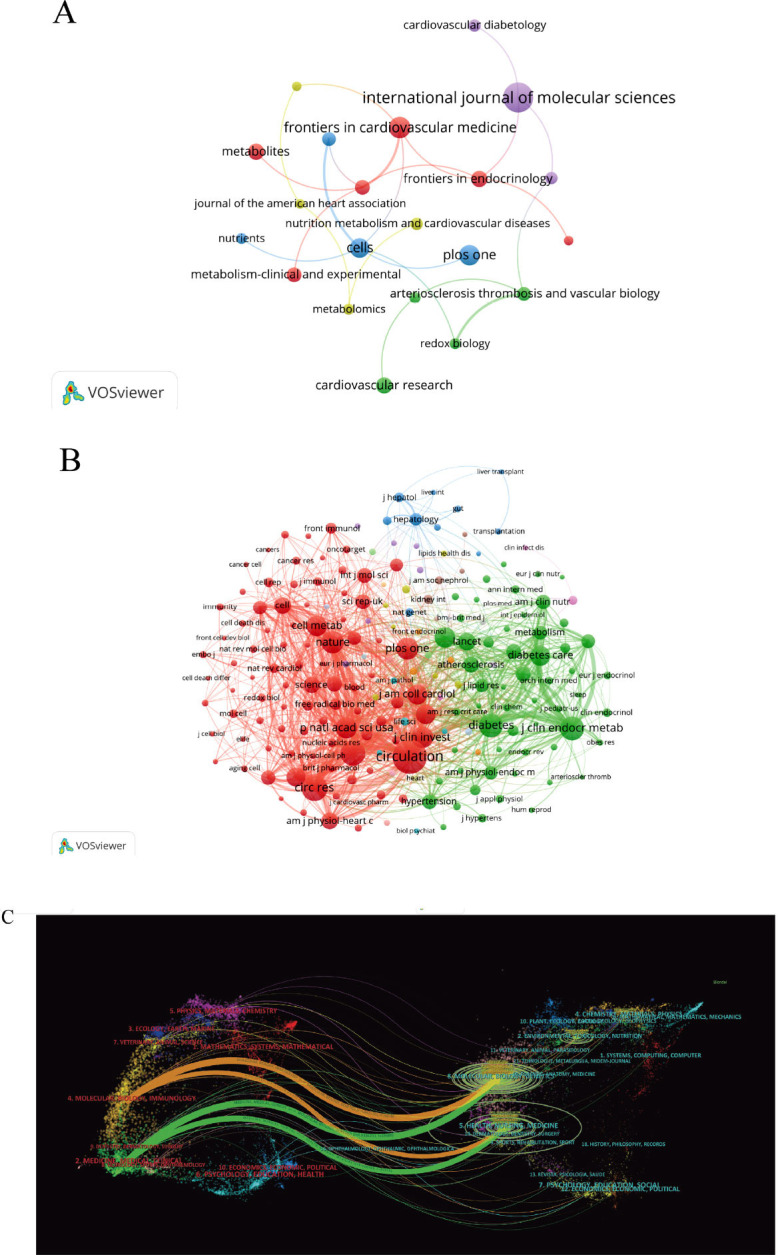
Visualizations and dual maps of journals generated by VOSviewer and CiteSpace. (**A**) A journal distribution map featuring journals with at least five publications; (**B**) A co-citation network of journals with at least 200 co-citations; (**C**) A dual-map, with citing journals on the left and cited journals on the right.

Analysis conducted through VOSviewer from 2000 to 2024 identified 409 SCI journals contributing to the literature, with 25 journals publishing five or more relevant articles (Fig. **5A** and Table **S2**). Among these, the *International Journal of Molecular Sciences* emerged as the top contributor with 23 articles, followed by *Frontiers in Cardiovascular Medicine* and *PLOS ONE*, with 14 and 13 articles, respectively. The journal *Cardiovascular Diabetology* holds the highest citation count (1,138) despite its publication volume not ranking among the top ten. It is followed by the *International Journal of Molecular Sciences* (901 citations) and *PLOS ONE* (662 citations). Furthermore, the *Journal of Clinical Endocrinology & Metabolism* had the highest average citations per article (57.9), followed by the *American Journal of Physiology - Heart and Circulatory Physiology* (52.3). Noteworthy is the fact that among the top ten journals with the highest publication counts, six are affiliated with institutions in Switzerland, three in the U.S., and one in the United Kingdom.

In our in-depth analysis of co-cited SCI journals, we identified three distinct clusters within the co-citation network, corresponding to the color-coded clusters displayed in Fig. (**5B**). Consequently, *Circulation* (1,822 citations), *Circulation Research* (1,072 citations), and the *Journal of Biological Chemistry* (1,028 citations) are the top three most frequently cited journals (Table **S3**). The red cluster primarily includes journals related to CVD. The green cluster mainly comprises metabolism-related journals, while the blue cluster includes journals focusing on liver metabolism. These journals highlight fundamental and clinical research pertaining to CVD and metabolism. They are cited to review existing studies and offer theoretical and empirical backing for our research.

The following presents an overlay of dual plots to analyze citation patterns across different research fields related to metabolic reprogramming in CVD (Fig. **5C**). Each node represents a journal, with citation relationships depicted by colorful curves flowing from left to right, providing a comprehensive visualization of the citation context. The figure highlights prominent green and orange citation paths: the orange paths represent molecular biology and immunology journals, which are frequently cited by journals focusing on health, nursing, and medicine. The green paths indicate that clinical and pharmaceutical journals are often cited by those in health, nursing, genetics, and molecular biology. This phenomenon illustrates significant cross-disciplinary citation patterns, where foundational research in molecular biology and immunology serves as a critical basis for advancements in clinical medicine and drug development. In turn, research in clinical and pharmaceutical journals extends into practical applications through health and nursing journals. These cross-disciplinary citations underscore the close relationship between basic and applied research, emphasizing the importance of interdisciplinary collaboration in driving scientific progress and addressing real-world challenges.

### 3.5 Analysis of the Most Co-cited References and the Most Frequently Occurring Keywords

**Fig. (6):**
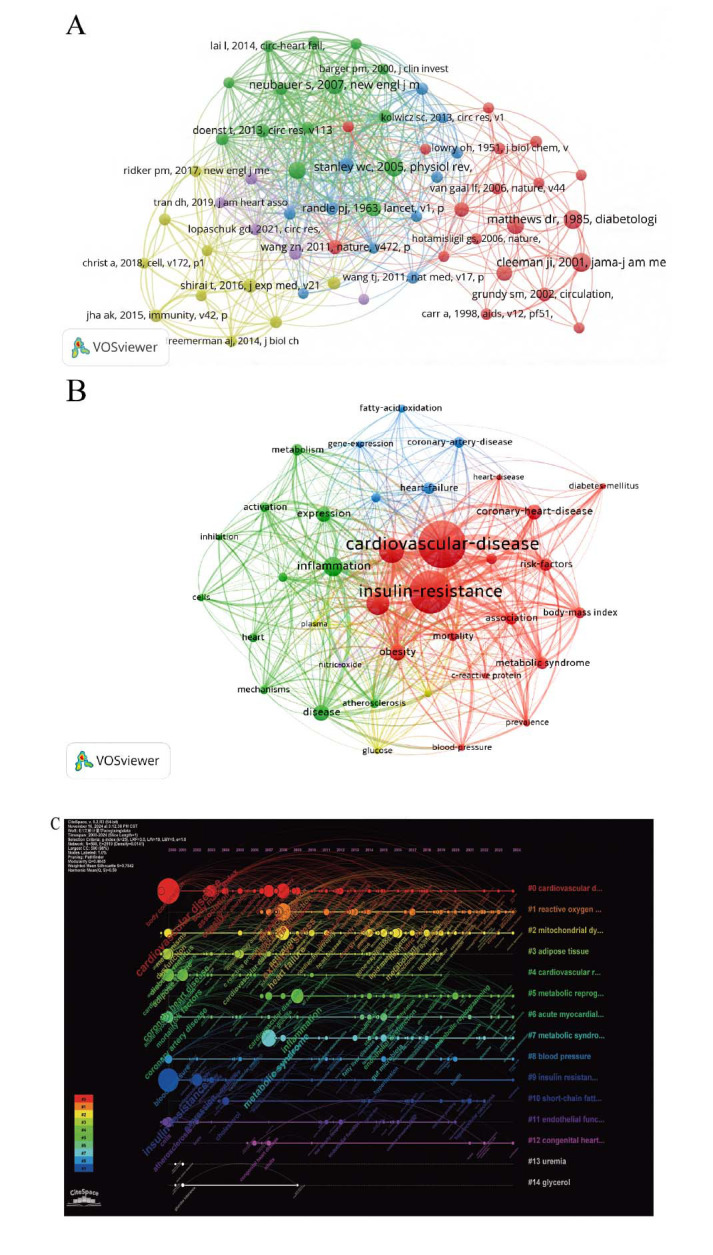
Visual maps of co-cited references and keywords generated through VOSviewer. (**A**) Displays a network of references co-cited 100 times or more; (**B**) Presents a co-occurrence network of keywords appearing 50 times or more; (**C**) The timeline view of keywords from publications.

To enable a more detailed co-citation analysis of references, VOSviewer was used to generate the top ten most-cited references between 2000 and 2024 (Fig. **6A** and Table **S4**). The most cited paper (29 times) is Executive Summary of The Third Report of The National Cholesterol Education Program Expert Panel on Detection, Evaluation, And Treatment of High Blood Cholesterol In Adults (Adult Treatment Panel III), published in 2001 in JAMA. The second most cited work is Myocardial substrate metabolism in the normal and failing heart by Stanley William C, published in 2005 in Physiological Reviews. It has been cited 28 times. The third is Homeostasis model assessment: insulin resistance and beta-cell function from fasting plasma glucose and insulin concentrations in man, published by Matthews DR in 1985 in Diabetologia, with 28 citations.

Additionally, we created a keyword co-occurrence network view of 765 documents through VOSviewer, where 37 keywords with a frequency of ≥20 were selected for visualization (Fig. **6B**). In the keyword co-occurrence network, the term Cardiovascular Disease itself serves as the central keyword with the largest node size. To enhance the clarity of keyword details, we also created a table of high-frequency keywords (individual occurrence frequency ≥ 46) (Table **3**). The table reveals that, in addition to the central keyword, terms such as Insulin Resistance, Oxidative Stress, Risk, and Inflammation also appear with high frequency.

The timeline visualization effectively demonstrates the dynamic evolution of research hotspots represented by keywords over time, enabling an exploration of the temporal characteristics and the rise and fall of research foci in the field (Fig. **6C**). Nodes and links in the map represent various keywords and their connections, whereas larger nodes indicate higher keyword frequency. Different clusters, distinguished by color, highlight specific themes: Cluster #0: Cardiovascular Diseases; Cluster #1: Reactive Oxygen Species; Cluster #2: Mitochondrial Dysfunction; Cluster #3: Adipose Tissue; Cluster #4: Cardiovascular Risk; Cluster #5: Metabolic Reprogramming; Cluster #6: Acute Myocardial Infarction; Cluster #7: Metabolic Syndrome; Cluster #8: Blood Pressure; Cluster #9: Insulin Resistance; Cluster #10: Short-Chain Fatty Acids (SCFAs); Cluster #11: Endothelial Function; Cluster #12: Congenital Heart Disease; Cluster #13: Uremia; Cluster #14: Glycerol. An analysis of keywords over time reveals the following trends: 2000-2010: Keywords during this period predominantly included “coronary heart disease”, “lipids”, “cardiovascular risk factors”, “cholesterol”, “cardiac dysfunction”, and “glucose tolerance”. Research themes were primarily centered on CVD risk factors, with a focus on metabolic issues such as lipid metabolism abnormalities, hypercholesterolemia, and impaired glucose tolerance. These studies emphasized early detection, prevention, and management strategies for metabolic disturbances affecting cardiovascular health. From 2011-2017 following keywords were highlighted during this phase, “microRNA”, “microbiota”, “DNA methylation”, “endothelial dysfunction”, “oxidative damage”, and “inflammation”. Research during this period shifted towards molecular mechanisms and cellular dysfunction in CVD, particularly examining aberrant regulation of molecular signaling pathways, epigenetic modifications, and cellular dysfunctions like oxidative stress and inflammatory responses that promote cardiovascular pathology. From 2018-2024 following eywords such as “macrophage polarization”, “substrate metabolism”, “ketone bodies”, “glutamine”, “glycolysis”, “branched-chain amino acids”, and “mass spectrometry” reflected a shift in research focus toward immunometabolism and metabolic reprogramming. This period placed significant emphasis on the functional changes in immune cells (*e.g.*, macrophages) under varying metabolic states. Immune cells adapt to environmental stimuli by modulating substrate metabolic pathways, such as glycolysis, ketogenesis, and glutamine metabolism, influencing inflammatory responses and the pathogenesis of CVD. These metabolic changes not only regulate immune cell functional phenotypes but also drive the reprogramming of metabolic pathways under pathological conditions, playing a pivotal role in disease progression.

### 3.6 Research Fundamentals and Emerging Hotspots

The figure below illustrates the citation bursts of the top 25 keywords in cardiovascular and metabolic research from 2000 to 2024, revealing the evolutionary trajectory of research hotspots in this field. As shown, the research focus has undergone three major phases:

**Early Phase (2000-2010):** This period primarily centered on the epidemiological characteristics and risk factors of CVD, with keywords such as “coronary heart disease” and “cardiovascular risk factors” dominating the research themes.

**Mid Phase (2010-2020):** Research focus shifted towards cellular phenotypic functions and inflammatory biomarkers, with keywords such as “C-reactive protein”, “endothelial dysfunction”, and “mitochondrial dysfunction” emerging as key topics.

**Fig. (7):**
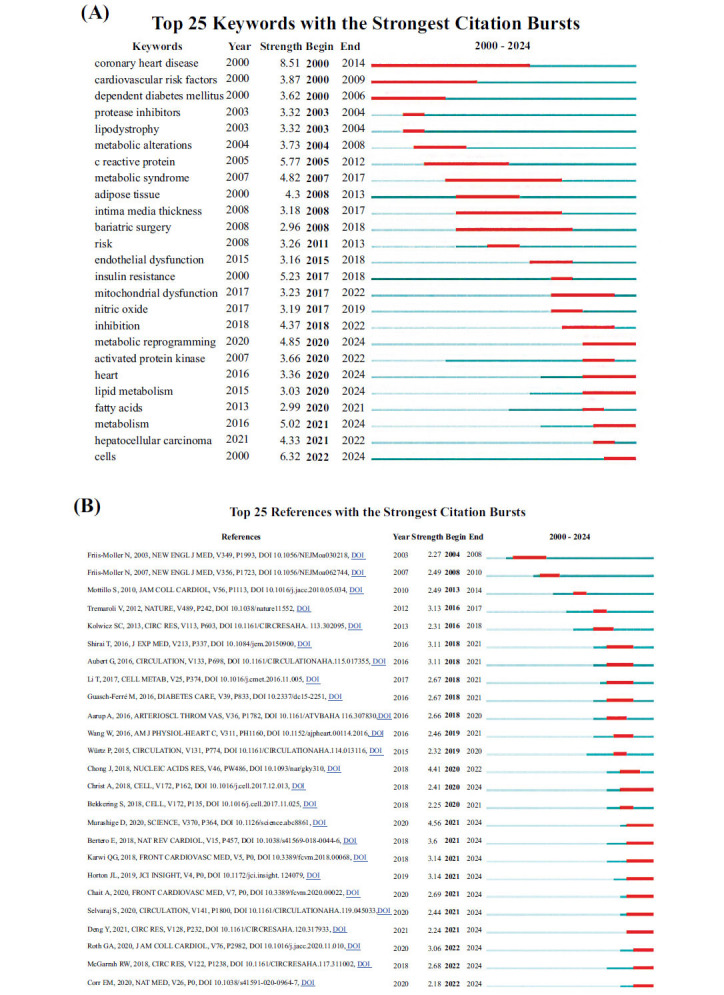
Analysis of literature related to CVD and metabolic reprogramming. (**A**) Top 25 keywords with the strongest citation bursts; (**B**) Top 25 articles with the strongest citation bursts.

**Recent Phase (2020-2024):** Keywords like “lipid metabolism”, “fatty acids”, “metabolism”, and “cells” have shown strong citation bursts, indicating a growing emphasis on metabolic reprogramming. Specifically, studies have focused on the metabolism of particular nutrients, such as lipids and fatty acids, and their role in modulating cellular functions, which in turn influence the pathogenesis of CVD. This reflects a shift toward these research areas as frontiers in the field (Fig. **7A**). Following this, detailed information on the top 25 articles with the strongest citation bursts is presented. The study revealed that the article by Fris-Møller N., published in 2003 in *The New England Journal of Medicine*, exhibited a burst intensity of 2.27 and experienced a notable citation surge between 2004 and 2008. Similarly, the 2015 article by Würtz P. showed a burst intensity of 2.32 during 2019-2020. Analysis of these data reveals that certain articles maintained their influence over many years, even extending into 2024, underscoring their enduring impact. These articles have not only provided critical theoretical foundations but also driven further advancements in the field (Fig. **7B**).

## 4 DISCUSSION

Bibliometrics is a statistical and mathematical method used for quantitatively analyzing publications across all scientific disciplines. It also has significant applications in the medical field, where it assesses the academic impact of medical research and reveals international collaboration patterns by analyzing publication volume, citation frequency, and team collaborations. By examining keywords frequencies and citation bursts, bibliometrics helps identify research hotspots, discover potential clinical applications, and uncover underexplored areas. This approach provides theoretical support for medical decision-making, clinical guideline development, and resource allocation [17-19]. In this study, bibliometric analysis was employed to investigate the development trajectory, key research areas, and intricate international cooperation networks in the field of metabolic reprogramming and CVD research from 2000 to 2024. Using tools such as VOSviewer and CiteSpace, we identified core authors, countries, institutions, and journals pivotal to this domain. Additionally, we conducted an in-depth analysis of the evolution of research topics and emerging hotspots.

Metabolic reprogramming refers to the cellular process of adapting metabolic pathways to meet altered energy demands and functional requirements in response to environmental changes or pathological states. This reprogramming typically involves a redistribution of energy metabolism to support cellular survival, proliferation, or differentiation. Over the past decade, the field of metabolic reprogramming has advanced rapidly, contributing significant insights into various diseases [20, 21].

### 4.1 Development Trend

In this study, the application of metabolic reprogramming in CVD research was analyzed using bibliometric methods. The analysis revealed that, from 2000 to 2006, the annual number of publications remained relatively stable. However, a gradual increase in research output was observed from 2007 onwards, culminating in a marked surge between 2021 and 2024, with a peak of 83 articles published in 2023. This trend highlights the growing focus and expansion of metabolic reprogramming research in the CVD field. Based on the findings of this bibliometric analysis, it is anticipated that future research on metabolic reprogramming in the context of CVD will continue its upward trajectory. As global scientific collaboration strengthens and data-sharing practices expand, research in this area is likely to accelerate. This progress holds promise for advancing the clinical translation of metabolic regulation therapies and gene-based treatments, ultimately enhancing the precision and effectiveness of CVD interventions [22, 23].

### 4.2 Author and Journal Analysis

Després, J.P., Silvia Fargion, and Hong Wang are the most prolific and highly cited authors in this field, each having published five papers. Among them, Després, J.P. has been cited 550 times, underscoring their significant influence in the domain. Furthermore, co-cited authors Lopaschuk, G.D. and Grundy, S.M. hold prominent positions, with 79 and 60 co-citations, respectively, highlighting their teams’ substantial academic impact in the study of metabolic reprogramming and CVD. Journal analysis revealed that between 2000 and 2024, 25 of the 409 journals included in the dataset published five or more relevant articles. Switzerland's *International Journal of Molecular Sciences* ranked first in publication volume, followed by Switzerland's *Frontiers in Cardiovascular Medicine* and the United States' *PLOS ONE.* However, the journal with the highest citation count was the United States' *Circulation,* emphasizing its central role in disseminating high-impact research in this area. These findings underscore the critical importance of these journals and provide a theoretical foundation for future investigations. Additionally, cross-references among journals in this field illustrate a dynamic interplay between basic and clinical research. Foundational disciplines provide the theoretical underpinnings for clinical applications, while applied clinical research, in turn, raises new questions and directions for basic science. This bidirectional feedback mechanism fosters a seamless integration of fundamental research and clinical practice, accelerating the translation of theoretical advancements into practical solutions. Ultimately, this synergy serves as a crucial driver for scientific progress in the field and contributes to the refinement of disease management strategies.

### 4.3 Global Cooperation and Regional Contributions

By examining the publication outputs of different countries and regions, we identified significant disparities in research performance and collaboration models in the field of metabolic reprogramming and CVD. These variations reflect differences in the allocation of scientific resources, policy priorities, and international collaboration strategies among nations [24]. The level of investment and support in specific disease areas directly influences a country’s academic output, while the structure and reach of scientific collaboration networks shape their global research standing. Collectively, these distinct approaches have synergistically driven the global advancement of metabolic reprogramming and CVD research [25]. Research in this domain has spanned 66 countries, 35 of which have published at least five papers. Among them, the USA leads with the highest number of publications and total citations, affirming its dominant role in this field. Italy and China follow, underscoring their growing prominence. Notably, while Canada has a relatively small number of publications, it boasts the highest average citation rate, indicating exceptional research quality. Similarly, Germany and the United Kingdom exhibit strong performance in terms of average citation impact. In terms of institutional influence, key players such as the University of Milan, ISCIII (Instituto de Salud Carlos III), and the University of Cambridge occupy central positions in international collaboration networks. Overall, the United States, China, and Italy emerge as the principal hubs of scientific research activity in this area. While European and North American countries maintain the highest research output collectively, Asian countries contribute comparatively fewer publications, highlighting an area for potential growth and investment. These findings underscore the pivotal role of international collaboration in shaping the trajectory of metabolic reprogramming and CVD research. Aging, Western diets, and COVID-19 significantly exacerbate metabolic reprogramming through shared mechanisms such as insulin resistance, inflammation, and oxidative stress, thereby promoting the onset and progression of CVD. Aging is associated with decreased insulin sensitivity, mitochondrial dysfunction, and chronic low-grade inflammation, which contribute to metabolic dysregulation and accelerate atherosis (AS). Western diets, characterized by high fat and sugar intake, increase the accumulation of toxic lipid intermediates, such as diacylglycerols and ceramides, further aggravating endothelial dysfunction and metabolic imbalance [26]. COVID-19 intensifies insulin resistance and metabolic abnormalities through cytokine storms and oxidative stress, significantly elevating the risk of CVD [27, 28]. The impact of these factors on metabolic reprogramming has been particularly evident in high-research-output countries (*e.g.,* the United States, China, and Italy). However, there remains significant potential for related studies in regions such as Asia. Strengthening international collaboration networks, especially through cross-national cooperation centered around key academic hubs, holds promise for further elucidating these mechanisms and developing targeted intervention strategies.

### 4.4 Keywords for Research Topics

The analysis of keyword co-occurrence networks and high-frequency keywords highlights enduring research hotspots and core issues in the field. High-frequency keywords such as *“Insulin Resistance”, “Oxidative Stress”, “Risk”*, and *“Inflammation”* reflect the persistent focus on metabolic disorders, oxidative states, disease risk factors, and inflammation-related mechanisms. These keywords not only exhibit strong associations with the central terms but collectively define the primary research framework in this domain. Insulin resistance plays a pivotal role in the onset and progression of CVD through metabolic reprogramming. It disrupts glucose and lipid metabolism, resulting in decreased glucose uptake and dysregulated fatty acid metabolism. These alterations shift cellular metabolism from oxidative to non-oxidative pathways, leading to the accumulation of toxic metabolites such as lactic acid and diacylglycerol. This metabolic reprogramming exacerbates endothelial dysfunction by impairing NO production and increasing the release of pro-coagulant factors, thereby fostering platelet aggregation and promoting AS.

Additionally, insulin resistance differentially impacts the PI3K and MAPK signaling pathways. While the PI3K pathway becomes impaired, leading to metabolic dysfunction, the MAPK pathway remains active, driving mitogenic effects. The MAPK and PI3K/Akt signaling pathways play a critical role in regulating endothelial Tight Junctions (TJs) and barrier function. Dysregulation of these pathways leads to endothelial cell damage and accelerates the development and progression of AS. The MAPK family (ERK1/2, p38, JNK, *etc*.) modulates the expression and localization of TJs proteins such as occludin, ZO-1, and claudin-5, exacerbating endothelial barrier permeability in response to inflammation and oxidative stress. The PI3K/Akt signaling pathway can enhance vascular barrier integrity by upregulating TJ proteins such as ZO-1, claudin-5, and occludin. However, under pathological conditions, it interacts with factors such as ROS, NF-κB, and Snail to promote TJs degradation and barrier disruption, leading to endothelial barrier dysfunction and accelerating AS formation [29-34]. Therefore, the interplay between PI3K dysfunction and MAPK activation synergistically accelerates the progression of AS, highlighting the intricate mechanisms through which insulin resistance contributes to CVD pathophysiology [35, 36].

Insulin resistance interacts intricately with gut microbiota imbalance and microRNAs to drive metabolic reprogramming, thereby promoting the onset and progression of CVD [37-39]. Gut microbiota dysbiosis contributes to systemic inflammation by producing lipopolysaccharide (LPS), which activates the TLR4-NF-κB pathway. This process reduces beneficial metabolites, such as short-chain fatty acids, while increasing harmful metabolites, like trimethylamine N-oxide, leading to endothelial barrier dysfunction, metabolic dysregulation, and AS [40, 41]. As molecular switches, microRNAs regulate key pathways by targeting insulin signaling (*e.g.*, miR-103 and miR-107 suppress IRS-1), inflammatory cascades (*e.g.*, miR-21 and miR-146a activate NF-κB and MAPK pathways), and lipid metabolism (*e.g.*, miR-33 inhibits ABCA1-mediated cholesterol efflux), thereby exacerbating insulin resistance and metabolic imbalance [42-46]. Additionally, gut microbiota-derived metabolites influence the expression of microRNAs, while microRNAs modulate gut barrier integrity by targeting genes such as ZO-1 and occludin, thereby shaping microbiota composition and function. This bidirectional interaction establishes a vicious cycle between metabolic and inflammatory pathways, driving physiology alterations in blood vessels [47]. These insights highlight the potential of probiotics to modulate gut microbiota and microRNA-targeted therapies as innovative strategies to mitigate CVD progression.

Oxidative stress plays a critical role in the pathological progression of CVD by driving the excessive accumulation of reactive oxygen species (ROS) and reactive nitrogen species, which not only disrupt cellular homeostasis but also trigger metabolic reprogramming, thereby establishing intricate pathological networks. ROS are predominantly generated by complexes I and III of the mitochondrial electron transport chain. Additional sources include NADPH oxidase, monoamine oxidase, and xanthine oxidase, whose activities are markedly upregulated under hypoxic conditions, further amplifying oxidative stress and exacerbating cellular damage [48, 49]. ROS can inflict direct damage on mitochondrial membranes, proteins, and DNA, with mitochondrial DNA (mtDNA) being particularly vulnerable. Unlike nuclear DNA, mtDNA exhibits a heightened susceptibility to oxidative damage due to its proximity to the electron transport chain, lack of protective histones, and limited repair capacity. This vulnerability results in mitochondrial dysfunction and perpetuates a cycle of oxidative stress, further exacerbating cellular injury [50]. Under hypoxic conditions, ROS levels rise abnormally, serving as critical regulators of metabolic pathways, including glycolysis, lipid metabolism, and energy production. This regulation is mediated through the activation of hypoxia-inducible factor (HIF) signaling, which orchestrates cellular adaptations to oxygen deprivation and reshapes metabolic processes to meet altered energy demands [51, 52]. For example, ROS induces increased expression of HIF-2α, which in turn promotes vascular remodeling and the development of pulmonary arterial hypertension (PAH) by upregulating SNAI that induces endothelium-mesenchymal transdifferentiation [52, 53]. Additionally, ROS facilitates the internalization of sodium-potassium ATPase by activating AMP-activated protein kinase. This process reduces cellular oxygen consumption, enabling metabolic adaptation to hypoxic conditions and promoting cellular survival under oxygen-deprived environments [54, 55]. Under hypoxic conditions, mitochondrial ROS stimulates upregulation of glycolysis, facilitating a shift toward rapid, anaerobic energy production to meet immediate cellular demands. However, the prolonged suppression of oxidative phosphorylation disrupts energy homeostasis, ultimately precipitating an energy crisis and impairing cellular function [48, 52]. Disorders of lipid metabolism are another significant feature. ROS induces lipid peroxidation to produce oxidation products (such as maleic aldehyde), exacerbating inflammation, atherosclerosis, and endothelial cell dysfunction [48, 56]. Furthermore, to preserve redox homeostasis, ROS elevates the demand for glutathione synthesis, depleting essential precursor amino acids such as cysteine in the process. This depletion disrupts amino acid availability, further perturbing cellular metabolic pathways and exacerbating metabolic stress [57]. Although ROS fulfill critical physiological roles in signal transduction, regulation of vascular tone, and oxygen sensing, their excessive production can have deleterious effects. Overabundant ROS can initiate inflammatory cascades, induce apoptosis, and cause tissue damage, thereby accelerating the progression of CVD and exacerbating their pathological outcomes.

In recent years, as the pivotal role of inflammatory mechanisms in CVD has become increasingly apparent, research has shifted its focus toward the activation of inflammasomes, particularly NLRP3 inflammasome, and their involvement in the pathogenesis of atherosclerosis, myocardial ischemia-reperfusion injury, and heart failure. This growing interest underscores the significance of inflammation as a central driver in the progression of cardiovascular diseases [58]. The NLRP3 inflammasome is a multiprotein complex that serves as a critical mediator in the pathogenesis of cardiovascular conditions, including atherosclerosis, myocardial ischemia-reperfusion injury, and heart failure. Its activation underscores its pivotal role in driving inflammatory responses that contribute to disease progression [59]. The activation of the NLRP3 inflammasome involves a meticulously regulated two-step process: a priming signal and an activation signal. The priming signal is typically initiated by pathogen-associated molecular patterns or damage-associated molecular patterns, such as high-mobility group proteins B1or heat shock proteins. These signals activate the NF-κB pathway *via* receptors like TLR4, leading to the upregulation of NLRP3, pro-IL-1β, and pro-IL-18. The subsequent activation signal is triggered by stimuli such as oxidative stress, extracellular ATP release, and the presence of uric acid or cholesterol crystals. These factors drive the assembly of the NLRP3 inflammasome by promoting the interaction of NLRP3 with ASC (apoptosis-associated speck-like protein containing a CARD) and Caspase-1. This process involves key mechanisms, including P2X7 receptor activation and lysosomal disruption. Activated Caspase-1 then cleaves the precursors of IL-1β and IL-18 into their mature, bioactive forms, which are secreted extracellularly. These cytokines amplify the inflammatory response, playing a critical role in the progression of inflammation-related pathologies [60-63]. Metabolic reprogramming and inflammation are intricately interconnected, forming a bidirectional regulatory relationship. During the inflammatory response, cells rapidly adapt to the heightened demand for energy and metabolic substrates by enhancing glycolysis, reprogramming fatty acid metabolism, and altering amino acid metabolism. Concurrently, metabolic byproducts such as succinate, lactate, and ROS act as signaling molecules, activating inflammatory pathways and further amplifying the inflammatory response. Chronic inflammation is tightly associated with metabolic disorders, including obesity, diabetes, and atherosclerosis. The interplay between inflammation driven by metabolic dysregulation and metabolic imbalances perpetuated by inflammation constitutes a fundamental mechanism underlying these conditions. This reciprocal interaction not only exacerbates disease progression but also highlights the intricate crosstalk between metabolic and inflammatory pathways in the pathogenesis of metabolic diseases [64-67]. Targeting glycolysis, fatty acid metabolism, and energy-sensing pathways has emerged as a promising therapeutic strategy for inflammatory diseases. These interventions offer innovative approaches to modulate the intricate interplay between metabolic processes and inflammation, paving the way for more effective treatments and deeper insights into the underlying mechanisms of these complex disorders.

### 4.5 Evolution of Research Trends

The integration of dual-map overlays and journal analysis of keyword trends reveals a gradual shift in research priorities, transitioning from macro-level disease management to the exploration of molecular mechanisms regulating signaling pathways and micro-level cellular metabolism. The evolution of research in the field of CVD and metabolism can be categorized into three distinct phases:

**Early Stage (2000-2010): Macroscopic Risk Factors, and Epidemiological Studies.** During this period, research primarily centered on the epidemiological characteristics of CVD and the identification of major risk factors. By examining the impact of metabolic disturbances-such as dyslipidemia and hypercholesterolemia-on cardiovascular health, early detection and management strategies were developed. The focus was on broad, population-level disease prevention and intervention, emphasizing risk factor assessment at the macro scale.

**Mid-Stage (2011-2017): Molecular Pathological Mechanisms and Cellular Dysfunction.** As research progressed, attention shifted to molecular mechanisms and cellular dysfunction underlying CVD. This phase delved into how intracellular pathological processes, including aberrant signaling pathways, epigenetic modifications, and inflammatory responses, drive disease progression. By uncovering these key molecular and cellular mechanisms, this stage laid the theoretical groundwork for identifying therapeutic targets and provided a more detailed understanding of the pathological changes associated with CVD.

**Late Stage (2018-2024): Cross-Disciplinary Research on Immunity and Metabolism.** Recent years have witnessed a growing emphasis on the intersection of immune metabolism and metabolic reprogramming. Research in this phase focuses on how metabolic networks regulate immune cell functions and their contributions to CVD. After AS or myocardial infarction (MI), a large number of monocytes are recruited to the damaged area, a process that supports their energy demands through HIF-1α-driven glycolysis. Additionally, neutrophils exacerbate local inflammation by forming neutrophil extracellular traps and activating the NLRP3-IL-1β axis, which further promotes IL-1β secretion and inhibits repair after the injury [68, 69]. In myocardial ischemia/reperfusion (I/R) injury, CCR2^+^ myocardial-resident macrophages promote the recruitment and infiltration of CCR2^+^ monocytes *via* the MyD88-dependent pathway, sustaining a strong inflammatory response that exacerbates myocardial damage and contributes to the malprogression of left ventricular remodeling [70]. The role of the glycolytic enzyme PKM2 in MI/R injury is closely associated with macrophage inflammatory responses. In LPS-induced M1 macrophages, PKM2 regulates the expression of pro-inflammatory cytokines such as IL-1β and IL-6 by activating HIF-1α and phosphorylating STAT3, thus maintaining the LPS-induced inflammatory storm. ISB, which targets PKM2 in macrophages, significantly improves cardiac function, reduces myocardial cell apoptosis, and suppresses the inflammation caused by reperfusion, making it a promising new drug for protecting the heart from MI/R injury [71]. Furthermore, PKM2's role in dilated cardiomyopathy (DCM) primarily affects cardiac function by regulating cardiomyocyte metabolism and transcription factor stability. In Jmjd4-deficient cardiomyocytes, PKM2 accumulation leads to metabolic dysregulation and mitochondrial dysfunction, triggering DCM. Jmjd4 mediates PKM2 degradation through interaction with Hsp70, maintaining metabolic homeostasis. In the absence of Jmjd4, PKM2 activity increases, leading to cardiac metabolic disorders and heart failure. PKM2 also interacts with transcription factors such as GATA4, GATA6, and P53 in cardiomyocyte nuclei to regulate cell survival and apoptosis [72, 73]. T-cell metabolic reprogramming plays a critical role in the development and progression of hypertension. Different T cell subsets rely on specific metabolic pathways to perform their functions, with CD4+ effector T cells depending on glucose metabolism and Treg cells preferring fatty acid oxidation. In the immune response to hypertension, inhibiting the mTOR signaling pathway promotes Treg polarization and suppresses Th17 differentiation, which may help alleviate immune-mediated inflammation in hypertension. Moreover, the mTORC1 and mTORC2 inhibitors, rapamycin and PP242, effectively treat salt-sensitive hypertension by reducing inflammation and kidney damage, further supporting the potential therapeutic role of immunometabolic regulation in hypertension [74-77]. Under hyperlipidemic conditions, natural killer (NK) cells enhance the uptake of free fatty acids and activate the PPAR and downstream PTEN-AKT-mTOR/FOXO1 signaling pathways, thereby influencing IFN-γ production. Additionally, lipid-loaded dendritic cells (DCs) increase the expression of PD-L1, TGF-β1, and NKG2D ligands through ROS, thereby suppressing NK cell activity and forming a bystander inhibitory effect. Although inhibition of PPAR or CPT1α partially restores NK cell function, full recovery requires consideration of other metabolic pathways [9]. BOLA3 downregulation promotes metabolic reprogramming of pulmonary arterial endothelial cells by regulating mitochondrial glucose metabolism and glycine homeostasis, a metabolic shift that plays a key role in the development of PAH, particularly in the clinical subtypes of type 1 and type 3 PAH [78]. BOLA3 deficiency also leads to fatty acid accumulation, which increases the expression of PD-L1 and TGF-β1 in DCs, further inhibiting NK cell function and promoting PAH. Research has shown that fatty acids control endothelial cell proliferation by incorporating carbon derived from fatty acid metabolism into nucleotide synthesis, especially under stress conditions. This finding may further expand the understanding of the Warburg effect in PAH and reveal the cross-talk between fatty acid oxidation and glycine metabolism [78-80]. In conclusion, immune cells coordinate inflammation, gene regulation, cell function, and tissue repair through metabolic reprogramming, significantly influencing the progression of CVD. These metabolic pathways provide promising therapeutic targets for CVD treatment. Meanwhile, advancements in mass spectrometry have enabled the precise quantification of metabolites and a comprehensive elucidation of metabolic networks. Using high-precision mass spectrometry, researchers can accurately detect and quantify diverse metabolic products in complex biological samples, monitor dynamic changes in metabolic pathways, identify potential biomarkers, and gain deeper insights into the role of metabolic reprogramming in disease pathogenesis and progression [66, 69].

## 5 FUTURE PERSPECTIVES

Targeting metabolic reprogramming holds tremendous potential for the future treatment of CVD, particularly within the context of the complex pathological mechanisms where metabolic dysregulation and immune responses intertwine. Drugs such as metformin, by activating and improving glucose metabolism, show promise in alleviating conditions like diabetic cardiomyopathy and AS [81, 82]. Glutaminase 1 inhibitors, such as CB-839, when combined with verteporfin, have been shown to alleviate symptoms of PAH [83]. Glycolysis inhibitors like 3-bromopyruvate can dampen immune cell inflammatory responses by suppressing glycolysis, thus inhibiting myocardial remodeling after injury and delaying the onset of PAH [84, 85]. Krüppel-like factor 5 (KLF5) is a key transcription factor in CVD, playing a crucial role in the pathological processes of AS, abdominal aortic aneurysm (AAA), and coronary artery disease. By regulating inflammation, oxidative stress, mitochondrial function, and metabolic balance, KLF5 accelerates the development and progression of CVD. For instance, in AS, KLF5 modulates the expression of LINC00346 and miR-148a-3p, promoting the release of inflammatory factors and endothelial dysfunction [86]. In AAA, KLF5 activates eIF5a to maintain mitochondrial integrity and inhibit reactive ROS production, while its downregulation leads to mitochondrial fission and exacerbates vascular smooth muscle cell senescence [87-89]. Given its pivotal role in these pathological processes, KLF5 has emerged as an important therapeutic target for CVD. ML264, a small-molecule inhibitor of KLF5, has shown promising therapeutic potential by suppressing KLF5 expression and activity. Studies have demonstrated that ML264 reduces abnormal aortic smooth muscle cell proliferation and migration, alleviates inflammation and ROS overproduction, and improves endothelial dysfunction and metabolic imbalance, thereby slowing the progression of AS and other CVDs [88]. Notably, KLF5 exacerbates the pathological progression of ischemic brain injury in stroke by promoting inflammatory responses, oxidative stress, mitochondrial dysfunction, and neuronal apoptosis while also aggravating brain edema and infarct volume. ML264, by downregulating KLF5 expression and activity, effectively alleviates neurological deficits, suppresses inflammatory responses and neuronal apoptosis, and improves metabolic function in brain tissue, demonstrating potential neuroprotective effects and offering a novel therapeutic target and strategy for the precision treatment of ischemic stroke [90, 91]. Estrogen-related receptor ERRα enhances mitochondrial oxidative capacity and fatty acid utilization, improving cardiac function and alleviating symptoms of heart failure [92]. The KLF5 inhibitor ML264 affects GFPT2, which is involved in amino sugar synthesis, to improve myocardial function following MI [93]. Additionally, the rapidly advancing field of single-cell sequencing offers novel insights, enabling the precise delineation of immune cell metabolism at the single-cell level within the CVD context. This technology reveals metabolic shifts across different immune cell populations and their contributions to disease progression [94, 95].

With continuous optimization and data accumulation, single-cell sequencing is poised to enable precision medicine tailored to individual metabolic profiles, thereby paving the way for new therapeutic strategies in CVD treatment. This will ultimately provide more targeted, personalized interventions, improving clinical outcomes and transforming the management of CVD in the future.

## CONCLUSION

In summary, this study conducted bibliometric and visualization analysis of CVD and metabolic reprogramming research from 2000 to 2024 using CiteSpace, VOSviewer, and SCImago Graphica, based on data from the WOSCC database. It systematically examined the publishing pattern in the past 24 years, and the number of global publications was the highest in the USA, and the number of European and American countries was generally higher than that of Asian countries. The most prolific and highly cited authors in this field include Després, JP, Fargion, Silvia, and Wang, Hong. The journal *International Journal of Molecular Sciences* published the most articles, while *Cardiovascular Diabetology* garnered the highest citations, reflecting the significant contributions of these authors and journals to the field. In recent years (2018-2024), research on metabolic reprogramming in CVD has largely focused on immune cells, particularly the functional changes of macrophages under different metabolic states. Immune cells modulate various metabolic pathways, including glycolysis, ketogenesis, and lipid metabolism, to influence the disease microenvironment, thus affecting the progression of CVD. In the future, the development of immune cell-targeted drugs may become a key breakthrough in CVD treatment, while the application of single-cell sequencing will provide more precise insights into immune cell metabolic dynamics, leading to revolutionary advancements in understanding disease mechanisms and developing novel therapeutic strategies.

## Figures and Tables

**Table 1:** Top 10 authors in terms of number of articles published.

**Rank**	**Author**	**Country**	**Documents**	**Citation**	**AAC**	**TLS**
1	Després, JP	Canada	5	550	110	17
2	Fargion, Silvia	Italy	5	396	79.2	12
3	Wang, Hong	China	5	164	32.8	26
4	Bergeron, J	Canada	4	542	135.5	17
5	Fracanzani, An-na Ludovica	Italy	4	377	94.25	11
6	Netea, Mihai G	Romania	4	149	37.25	8
7	Riksen, Niels P	Netherlands	4	153	38.25	8
8	Mouton, Alan J	USA	4	220	55	19
9	Wang, Zhen	China	4	46	11.5	15
10	Andriantsitohai-na, Ramareson	France	4	288	72	7

**Abbreviations:** AAC: Average Article Citations; TLS: Total Link Strength.

**Table 2:** Top 10 countries in terms of number of articles published.

**Rank**	**Country**	**Documents**	**TC**	**AAC**	**TLS**
1	USA	189	11183	59.17	147
2	Italy	129	5875	45.54	75
3	China	125	2670	21.36	26
4	Spain	73	3085	42.26	69
5	Germany	50	3241	64.82	75
6	Canada	44	3920	89.09	28
7	England	40	2517	62.93	81
8	Brazil	40	823	20.58	28
9	Netherlands	32	1823	56.97	69
10	France	31	1524	49.16	42

**Abbreviations:** TC: Total Citations; AAC: Average Article Citations; TLS: Total Link Strength.

**Table 3:** Top 10 keywords in terms of number of articles issued.

**Rank**	**Keywords**	**Occurrences**	**TLS**
1	Cardiovascular Disease	168	321
2	Insulin Resistance	152	336
3	Oxidative Stress	88	190
4	Risk	82	185
5	Inflammation	69	180
6	Disease	57	122
7	Obesity	56	154
8	Expression	54	105
9	Coronary Heart Disease	54	103
10	Risk Factors	46	114

**Abbreviation:** TLS: Total Link Strength.

## Data Availability

All the data and supporting information is provided within the article.

## LIST OF ABBREVIATIONS

CVD: Cardiovascular Disease
AAA: Aortic Aneurysm
AS: Atherosis
DCM: Dilated Cardiomyopathy
DCs: Dendritic Cells
HIF: Hypoxia-inducible Factor
I/R: Ischemia/Reperfusion
KLF5: Krüppel-Like factor 5
LPS: Lipopolysaccharide
MI: Myocardial Infarction
mtDNA: Mitochondrial DNA
NK: Natural Killer
PAH: Pulmonary Arterial Hypertension
ROS: Reactive Oxygen Species
TJs: Tight Junctions
