# Supplementary material for: A Bibliometric and Visualization Analysis of Metabolic Reprogramming in Cardiovascular Diseases: Trends, Key Contributors, and Future Directions from 2000 to 2024
Source: Curr Cardiol Rev. 2025 Feb 7;21(4):E1573403X371021. doi: 10.2174/011573403X371021250109064231 (PMC12180361; doi:10.2174/011573403X371021250109064231)
Supplement: Supplementary file 1 [file CCR-21-4-E1573403X371021_SD1.pdf]

Supplementary Material

A Bibliometric and Visualization Analysis of Metabolic Reprogramming in Cardiovascular Diseases: Trends, Key Contributors, and Future Directions from 2000 to 2024

Xing Chen[1], Liu-Lin Yang[2], Li-Xiang Li[1] and Yan Deng[1,*]

[1]*The First Affiliated Hospital of Guangxi Medical University, Guangxi Medical University, Nanning, China;* [2]*Hepatological Surgery Department, The First Affiliated Hospital of Guangxi Medical University, Guangxi Medical University, Nanning, China*

Supplementary Table 1. Top 10 institutions in terms of number of articles published

Rank	Organization	Country	Documents	TC	AAC	TLS
1	Univ Milan	Italy	21	867	41.29	10
2	Inst Salud Carlos III	Spain	12	232	19.33	0
3	Univ Cambridge	Eng-land	11	784	71.27	5
4	Univ Naples Federico II	Italy	11	277	25.18	2
5	Univ Rovira & Virgili	Spain	11	263	23.91	1
6	Johns Hopkins Univ	USA	10	676	67.60	6
7	Univ Oxford	Eng-land	10	326	32.60	5
8	Harvard Med Sch	USA	10	191	19.10	3
9	Univ Palermo	Italy	10	188	18.80	1
10	Univ Laval	Can-ada	9	2235	248.33	5

TC: Total Citations; AAC: Average Article Citations; TLS: Total Link Strength.

Supplementary Table 2. Top 10 journals in terms of number of articles published

Ra-nk	Journal	Country	IF(2023)	JCR(2023)	Documen-ts	Citati-ons	AAC	TLS
1	International Journal of Molecular Scienc-es(IJMS)	Switzerla-nd	4.9	Q2	23	901	39.2	3
2	Frontiers in Cardiovas-cul-ar Medicine	Switzerla-nd	2.8	Q2	14	201	14.4	4
3	PLOS ONE	USA	2.9	Q1	13	662	50.9	1
4	Cells	Switzerla-nd	5.1	Q2	12	228	19	6
5	Frontiers in Endocri-nology	Switzerla-nd	3.9	Q2	9	140	15.6	3
6	Cardiovascul-ar Re-search	England	10.2	Q1	9	368		1
7	Metabolites	Switzerla-nd	3.4	Q2	9	71	7.9	1

8	Antioxidants	Switzerla-nd	6	Q1	9	136	15.1	0
9	American Journal of Physiology - Heart and Circulatory Physiology	USA	4.1	Q1	8	418	52.3	6
10	Journal of Clinical Endocrinology & Metabolism	USA	5	Q1	8	463	57.9	1

IF: Impact Factor; JCR: Journal Citation Reports; AAC: Average Article Citations; TLS: Total Link Strength.

Supplementary Table 3. Top 10 co-cited journals in terms of number of articles issued.

Rank	Journal	Country	IF(2023)	JCR(2023)	Citations	TLS
1	Circulation	USA	35.5	Q1	1822	170659
2	Circulation Research	USA	16.5	Q1	1072	123008
3	Journal of Biological Chemistry	USA	4	Q2	1028	116954
4	Journal of Clinical Endocrinology & Metabolism	USA	5	Q1	941	95628
5	Diabetes	USA	6.2	Q1	937	104086
6	Nature	England	50.5	Q1	829	109853
7	PLOS ONE	USA	2.9	Q1	802	73958
8	Journal of Clinical Investigation	USA	13.3	Q1	787	87223
9	Proceedings of the National Academy of Sciences of the Unit-ed States of America	USA	9.4	Q1	776	95353
10	New England Journal of Medicine	USA	96.2	Q1	732	65922

IF: Impact Factor; JCR: Journal Citation Reports; TLS: Total Link Strength.

Supplementary Table 4. Top 10 cited references

Rank	Title	Journals	Authors	Year	Citations	TLS
1	Executive Summary of The Third Report of The National Cholesterol Education Program (NCEP) Expert Panel on Detection, Evaluation, And Treatment of High Blood Cholesterol In Adults (Adult Treatment Panel III)	JAMA	JAMA	2001	29	31
2	Myocardial substrate metabolism in the normal and failing heart	Physiological reviews	Stanley Wil-liam C	2005	28	95
3	Homeostasis model assessment: insulin resistance and beta-cell func-tion from fasting plasma glucose and insulin concentrations in man	Diabetologia	Matthews D R	1985	28	16
4	The failing heart--an engine out of fuel	The New England journal of medicine	Neubauer Stefan	2007	27	92
5	Myocardial fatty acid metabolism in health and disease	Physiological reviews	Lopaschuk Gary D	2010	24	111
6	Banting lecture 1988. Role of insulin resistance in human disease	Diabetes	Reaven G M	1988	23	23

7	Estimation of the concentration of low-density lipoprotein cholesterol in plasma, without use of the preparative ultracentrifuge	Clinical chemistry	Friedewald W T	1972	20	17
8	The glucose fatty-acid cycle. Its role in insulin sensitivity and the metabolic disturbances of diabetes mellitus.	Lancet (London, England)	RANDLE P J	1963	19	75
9	Obesity is associated with macrophage accumulation in adipose tissue	The Journal of clinical investigation	Weisberg Stuart P	2003	17	30
10	The glycolytic enzyme PKM2 bridges metabolic and inflammatory dysfunction in coronary artery diseas	The Journal of experimental medicine	Tsuyoshi Shirai	2016	16	47

TLS: Total Link Strength
